# Co-Designing a Program to Lower Cardiovascular Disease Risk in Midlife Black Women

**DOI:** 10.3390/ijerph19031356

**Published:** 2022-01-26

**Authors:** Holly J. Jones, Tamilyn Bakas, Sheila Nared, Jacqueline Humphries, Julie Wijesooriya, Melinda Butsch Kovacic

**Affiliations:** 1 College of Nursing, University of Cincinnati, Cincinnati, OH 45267, USA; bakastn@ucmail.uc.edu; 2 West End Community Research Advisory Board, Cincinnati, OH 45214, USA; snnared@gmail.com (S.N.); Jacqueline.Humphries@cchmc.org (J.H.); butschms@ucmail.uc.edu (M.B.K.); 3 Seven Hills Neighborhood Houses, Cincinnati, OH 45214, USA; Julie.Wijesooriya@cchmc.org; 4 Cincinnati Children’s Hospital Medical Center, Cincinnati, OH 45229, USA; 5 College of Allied Health Sciences, University of Cincinnati, Cincinnati, OH 45267, USA

**Keywords:** community-based participatory research, intervention studies, cardiovascular diseases, heart disease risk factors, African American, women’s health services

## Abstract

Midlife Black women suffer disproportionately from heart disease and stroke in comparison to White women of similar age and demographic. Risk for cardiovascular disease (CVD) and stroke is largely considered to be modifiable yet CVD prevention and awareness campaigns have been less effective among Black women. Decreased awareness of personal CVD risk is associated with delays in the presentation of women to the emergency room or health care providers for symptoms of myocardial infarction. The Midlife Black Women’s Stress and Wellness (B-SWELL) program was designed to increase awareness about CVD risk factors, stress, and healthy lifestyle behaviors among midlife Black women. In partnership with an existing Community Research Advisory Board (C-RAB), materials were developed and culturally adapted for the B-SWELL program. Following successful development of the B-SWELL materials, a trial of the B-SWELL program was conducted with a sample of midlife Black women recruited from the community. The program was co-facilitated by members of the C-RAB. We outline the strategies used to successfully co-create and trial the B-SWELL program materials and reflect on the strengths and challenges associated with the development of a culturally tailored heart disease prevention program using community participatory methods.

## 1. Introduction

Midlife Black women in America have a 69% higher death rate from heart disease and double the death rate from stroke compared to White women [1]. It is estimated that 49% of adult Black women have some type of heart disease and 40% have hypertension, a major precursor to heart disease. Black women are also more likely to suffer from chronic medical conditions and disability or adverse outcomes resulting from such conditions [2]. Heart disease and many of its related conditions are largely modifiable and preventable through healthy lifestyle behaviors. National campaigns such as those supported by the American Heart Association (AHA), have failed to increase awareness of relative heart disease risk in women of color, particularly Black women [3,4]. Reasons proposed for the failure to improve CVD awareness in Black women include a lack of tailored and targeted awareness campaigns and limited access to cardiovascular screenings [5].

Heart disease was historically viewed as a ‘man’s disease’ yet there has been a significant rise in heart disease and cardiac events among women in recent decades [6]. In response to this rise, multiple awareness campaigns have been enacted to increase women’s knowledge and awareness of heart disease and its warning signs [7,8,9]. According to surveys conducted in 2002, awareness of heart disease risk was only 36% among Black women compared to 65% of White women [3]. Recent strides among Blacks have been threatened by a recent decline in heart disease awareness across all groups [4]. A lack of awareness is associated with delays in the presentation of women to the emergency room or health care providers for symptoms of myocardial infarction [10]. Lower rates of awareness are also linked to poor lifestyle behaviors, contributing to poor health outcomes and comorbidity. Disparities in the effectiveness of awareness campaigns may be related to socially constructed ideas about the meaning of heart disease and prevention [11]. The adoption of healthy lifestyle behaviors is a choice which may require targeted interventions that leverage cultural norms, beliefs, and behaviors [12].

Community participatory research is proposed to improve delivery of campaigns and programs targeted to high-risk groups such as midlife Black women [13]. Community-based research is grounded in social justice and thus, focuses on the integration of knowledge and expertise from community members and researchers to address major social issues such as health disparities [13,14]. Often researchers enter community research projects with a prescriptive attitude, discounting the expertise, experience, skills, and culture within the community of interest. Through community engagement, partnerships can be built, bridging gaps that would otherwise hinder communication, development of trust, and mutual respect. Community engaged research encourages equitable involvement on both sides, giving voice to those who stand to benefit most from the research and increasing the potential for sustainable solutions [15].

Despite its strengths, there remains a paucity of programs designed using participatory methods with high-risk populations such as midlife Black women. Fewer still are designed to serve as an adjunct to routine medical care; educating and empowering midlife Black women to become active, knowledgeable participants in the health care system. The purpose of this manuscript is to describe the successful pilot of the Midlife Black Women’s Stress and Wellness (B-SWELL) program designed to increase awareness about heart disease, risk factors, and healthy lifestyle behaviors among midlife Black women.

### Life’s Simple 7

The B-SWELL program uses the AHA’s Life’s Simple 7 (LS7) to highlight key lifestyle behaviors shown to lower heart disease risk. LS7 was introduced in 2010 to target key modifiable behaviors related to heart disease risk. The LS7 behaviors are exercise, losing weight, eating better, stopping smoking, controlling cholesterol, managing blood pressure, and reducing blood sugar [7]. LS7 was selected for this research because its metrics are easy to use and amenable to self-evaluation. Individuals can rate their performance for each key behavior using a scale of 0 (poor) to 2 (ideal). Since its inception, the LS7 metrics have been associated with lower risk for heart failure, diastolic dysfunction, stroke, and mortality related to CVD [16,17]. The LS7 metrics have also been used to assess for cancer risk, metabolic syndrome, and all-cause morbidity [18,19].

Despite its broad applications, adoption and adherence to the LS7 healthy lifestyle behaviors is far from simple. The adoption and maintenance of healthy lifestyle behaviors is of great interest when targeting midlife Black women who disproportionately suffer from chronic conditions such as hypertension, diabetes, and obesity that predispose them to CVD [19]. A recent systematic review focused on the use of community engaged and community participatory research to promote LS7 found only two studies that evaluated all seven of the LS7 metrics [20]. Most research is based on only one or two of the LS7 metrics, highlighting the challenges associated with addressing all seven [20]. Indeed, culturally tailored and targeted programs promoting heart disease awareness and adoption of the LS7 metrics are lacking for midlife Black women. Community participatory research methods in this original research were instrumental to the successful development, cultural adaptation, and trial of a tailored program for midlife Black women focused on the adoption of LS7 healthy lifestyle behaviors.

## 2. Materials and Methods

### 2.1. Program Conceptualization

The B-SWELL program was conceptualized as a program to leverage stress reduction and culturally appropriate strategies to increase the adoption of healthy lifestyle behaviors among midlife Black women. Development of the B-SWELL program is based on preliminary qualitative work that identified key stress-related themes and barriers faced by midlife Black women when adopting healthy lifestyle behaviors [21,22,23]. The first study identified six major stressors experienced by midlife Black women: finances, caring for family members, relationships, personal health and aging, race and discrimination, and raising children [22]. Those findings overlapped with the second qualitative study that identified four primary sources of stress: workplace, parenting, finances, and social media. Gendered racism and discrimination and life imbalance emerged as underlying stressors [21]. In both studies, focus groups were comprised of midlife Black women recruited from the community. These preliminary findings acknowledged unique stressors experienced by midlife women and the barriers they pose to the adoption of healthy lifestyle behaviors, supporting development of a culturally tailored program for midlife Black women.

### 2.2. Community Participatory Methodology and Setting

To incorporate community participatory methods into this research project, the principal investigator (PI) developed a working relationship with an existing, institutionally supported Community Research Advisory Board (C-RAB). The C-RAB is comprised of both male and female adolescent and adult community members (*N* = 15) aged 16 to 78 years old. Meetings between the PI and the C-RAB were initially held at a local community center but were later transitioned online given COVID-19 restrictions. The purpose of the C-RAB is multifold. The board seeks to (1) engage university researchers to ensure they support the health goals of the community in addition to collecting study data, (2) provide feedback on how to make research easier and more understandable for local minority communities, and (3) inform what research happens in their community. Board members are trained in research ethics and educated in various topics of health research. Each member keeps a binder with research training materials, reference documents, the C-RAB bylaws, and personal notes. C-RAB members are provided $30 for each C-RAB meeting in which they participate ($20/hr).

The PI gained entry with the C-RAB through a research mentor who had previously worked closely with the C-RAB membership over several years to increase their visibility and activities with community partners. The PI’s relationship with the C-RAB began two years prior to the start of the research project, cementing the partnership with its members. During that time, the PI attended routine monthly C-RAB meetings, participated in community-based events such as health fairs and health education workshops, and provided regular updates on her research activities. In attending routine meetings, the PI became familiar with the broad scope of C-RAB activities and supported their efforts by providing them her feedback and opinions when asked. The PI presented her research proposal, including its focus on LS7, to the C-RAB to assess interest and receive their critical responses. Once funding was secured, steps were taken to establish a formal partnership, specifying roles and duties for each the PI and the C-RAB members.

## 3. Results

### 3.1. Phase 1

#### 3.1.1. Partnership and Recruitment

A formal partnership was established between the PI and the C-RAB (Appendix A). The partnership agreement outlined the roles and responsibilities of the C-RAB and identified several goals which included (1) help with development of the B-SWELL program prototype materials, (2) input about recruitment, site locations, and marketing for the program, (3) feedback and recommendations about the relevance and use of the program outcomes, and (4) assistance with dissemination of the findings to the greater community (Table 1). The formal community partnership agreement also outlined the hourly wage that C-RAB members were to receive for hours worked on the project. To achieve the identified goals, one C-RAB member was selected to attend quarterly scientific advisory board meetings and a small working group of five C-RAB members was assembled to work closely with the PI in the development of the B-SWELL program prototype. Participation in the C-RAB working group was limited to adult Black women over the age of 40. Women meeting the age criteria were approached for participation and five women volunteered to participate. A long-term independent contractor’s agreement was established for each participant with the institution; solidifying their roles as community co-investigators and facilitating the payment processes. The rate of pay for C-RAB members in the institution’s contractor agreement was consistent with the formal partnership agreement. Members were responsible for tracking their time and submitting their hours for payment.

**Figure 1:**
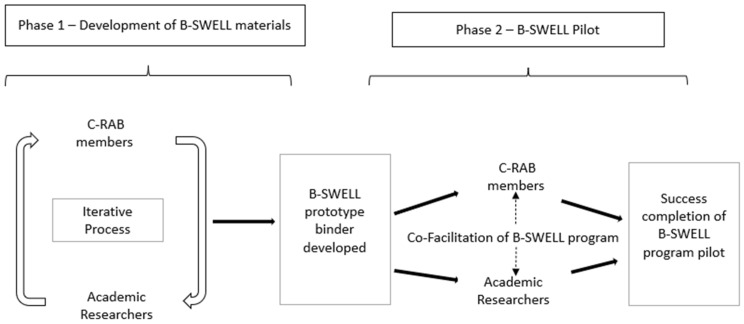
Collaborative research activities.

During phase 1 of this study, the PI worked closely with the C-RAB working group to develop the B-SWELL materials (Figure 1). This iterative process would span one year. As previously stated, the PI established a working relationship with the C-RAB prior to beginning phase 1 of this study. In this way, trust was established and the C-RAB members gained familiarity with this study’s purpose and expected outcomes. An initial outline for each module and its content was developed by the PI. Following development, the content was passed along to the C-RAB working group. Each member of the working group reviewed the materials, providing critique and recommendations for improvement or further development. Feedback focused on the informational content of the B-SWELL materials, clarity of the content, and cultural relevancy. Updates were provided intermittently to the larger C-RAB group to solicit feedback and discussion. This cyclical process was successful in the development and refinement of the B-SWELL content and materials.

#### 3.1.2. B-SWELL Materials

The final B-SWELL prototype consisted of eight modules containing culturally adapted content and materials. The first module, ‘Heart Disease and You’, provides an overview of CVD risk for midlife Black women, introduces the LS7 behaviors (exercise, eating better, losing weight, stopping smoking, managing blood pressure, controlling cholesterol, and reducing blood glucose), and discussing the relationship between stress and CVD. The remaining seven modules were dedicated to the LS7 healthy lifestyle behaviors and titled: ‘Reducing Heart Disease Risk with Exercise’, ‘Heart Disease and Nutrition’, ‘Heart Disease and Weight Loss’, ‘Heart Disease and Smoking’, ‘Blood Pressure and Heart Disease’, ‘High Cholesterol and Heart Disease’, and ‘Diabetes and Heart Disease’. Content in each module provided in depth information about the respective LS7 behavior and its relationship to CVD and stress to increase personal knowledge and awareness. Content was written at a 5th grade level to improve accessibility and ease of use. Modules also included a glossary of relevant terms, strategies and tips appropriate to the LS7 behavior being addressed, information about stress, tips for stress reduction, and case studies with sample goal setting forms highlighting key take-away ideas and relevant issues for midlife Black women. Content was strategically selected to enhance cultural receptivity (Table 2). The case studies were included at the end of each module to address issues relevant to midlife women and women of color. For example, in module 6, ‘Blood Pressure and Heart Disease’, the case study highlighted a hypothetical woman named ‘Brenda’ who had issues relevant to self-management of hypertension and struggles associated with grandparenting and juggling household expenses on a fixed income. Other case studies addressed issues like menopause, workplace discrimination, and racial stereotypes.

### 3.2. Phase 2

#### B-SWELL Pilot

Once the B-SWELL prototype was developed, the materials were validated, and an 8 week pilot trial was conducted. Two female midlife C-RAB members were invited to participate in the pilot as program facilitators because they had prior experience working with community-based programs and leading group activities. The C-RAB members participating in this phase of the research were bound by the partnership agreement described above but were also included in this study’s IRB as members of the research team. Study activities were approved by the Institutional Review Board of the University of Cincinnati (2019-0426) and registered on ClinicalTrials.gov (NCT04404478). Training of the C-RAB facilitators and other members of the research team took place prior to initiation of the pilot during weekly team meetings. The PI was also available for one-to-one instruction between the weekly meetings and throughout the pilot trial. Training included information about their role as facilitators, facilitation skills, study materials, data management, introduction to technology used during this study, and completion of self-evaluation forms (Table 3). Time was set aside to practice use of the technology and accessing the online virtual platform that would be used for the group meetings. Both women had prior experience attending online meetings; however, they were new to leading online meeting activities. The C-RAB facilitators were provided with a tablet device to use for the duration of the online B-SWELL pilot; the same tablet was provided to all participants in this study.

As facilitators, the primary responsibilities of the community researchers were to maintain the flow of the program; monitor participant engagement; create a safe environment in which each participant could speak, be heard, and feel comfortable sharing experiences; and promote the B-SWELL agenda. During the pilot trial, the C-RAB facilitators attended weekly team meetings to receive and give feedback, discuss observations, and voice concerns or ideas to improve the program. Facilitators were also given a guide that contained an ordered list of session activities and pre- and post-session checklists. Open-ended questions were included in the guide to encourage self-reflection and identify areas in need of improvement (Table 4). Sessions of the B-SWELL pilot were audio recorded. The principal investigator reviewed the audio recordings regularly to evaluate sessions facilitated by C-RAB members and provide feedback. In turn, C-RAB facilitators were asked to perform self-evaluations using the facilitator guide.

### 3.3. C-RAB Member Feedback

Following completion of the B-SWELL pilot study, interviews were conducted with members of the C-RAB working group and facilitators. The purpose of the interviews was to obtain feedback on the collaborative process used to develop and pilot the B-SWELL. Questions focused on the design process and facilitation. Interviews were conducted by an objective community liaison who did not participate in the study activities and audio recorded for the interviewer’s benefit only.

Four of the five working group members participated in interviews about the developmental process. During the developmental phase, paper copies of the modules were mailed to C-RAB members for feedback and returned in the same manner. With the written feedback in hand, the PI then conducted phone interviews to elicit more information. Regarding the development process, one member stated *“Really worked. Because [it] gave us time to go over it and think about it and then called us. Had ample time to go over it and give input”*. When asked if the process allowed them to contribute the way they wanted, the overall group repose was affirmative. Reponses included *“Definitely felt were heard because made the calls individually. She could focus on what we each individually had to say”* and *“She actually had their papers in front of her from each of us that she could refer to and ask further questions”*. C-RAB members were also asked if they felt their opinions and input were valued. Responses included *“[The PI] made us feel that we were really important. That what we said and did was so valuable to her to get the grant and move forward.”* and *“She wasn’t judgmental. Didn’t judge our answers. Just really listened to what we said and made us feel good.”* Finally, the C-RAB members were asked their opinion about the completed B-SWELL binder. The C-RAB members liked the information contained in the binder and were able to recall specific details that resonated with them. There were also suggestions to share the information such as *“It’s like everyone in the world should have this information”* and *“She definitely needs to take it somewhere else where she can present the whole binder and have others involved in it”.*

Interviews related to facilitation of the B-SWELL pilot were conducted with both community facilitators. Facilitator training took place prior to the pilot trial and weekly debriefing meetings were for the duration of the pilot. The facilitators were asked to evaluate their training and make suggestions for improvement. In general, the training was considered helpful. One facilitator stated that the training taught her *“How not to be biased in my thinking and responding. How to really hear what people were really saying.”* The other facilitator commented on the facilitation guide, stating *“[I] also liked that we had guide sheets, that helped to keep us on our time. And a list of all the women ahead of time.”* However, it was suggested that an evaluation midway into the B-SWELL trial would *“…Help us as we move forward and meet everybody’s needs.”* Additionally, timing of the facilitator meetings caused some confusion thus, it was suggested to hold separate meetings for each B-SWELL session rather than combining. The facilitators also offered suggestions to improve the delivery of the B-SWELL program. One facilitator suggested spacing the B-SWELL sessions. Another suggested longer sessions to have more time with participants, *“…sometimes wished [we] had another week, especially with the first group since they wanted more.”*

In essence, the C-RAB members expressed satisfaction with their participation in development of the B-SWELL materials and facilitation of the program. When sharing thoughts about their role in the development of the B-SWELL modules, one stated, *“She made you feel so good….like we were researchers.”* Others commented *“Really felt we were part of the project…Not just sitting down and telling us what she is going to do.”* and *“Felt honored. Felt special.”* In regard to the role as a community facilitator, one lady commented *“The engagement piece was really good–I really enjoyed that. All of us have something in common. Learn different stuff that may come up and helped me to pay attention to (health) stuff a little more.”* Another stated that she *“Felt valued. Learned a lot. Would have loved to have continued”* and *“It was good. Learned a lot. A lot of it applied to me as a Black woman. Helped you to know that others are going through the same stuff.”*

### 3.4. Strengths and Challenges

Strengths associated with this research are many. The enhanced relationships between the PI, C-RAB, and the institution cannot be underestimated. The community C-RAB members were able to participate in the research process on multiple levels, watching their ideas come to fruition. Ideas generated by the C-RAB members were authentic, embodying the essence of the community. For example, the C-RAB members suggested ideas for case studies that were later found to resonate greatly with participants during the B-SWELL pilot. One module described the reluctance of a midlife Black woman to see a health care provider due to fear. All participants gained experiential knowledge of the processes and time required to properly conduct community participatory research projects. Trust was established on all levels and instrumental in the willingness of the C-RAB members to remain engaged in the B-SWELL project. Mutual respect was earned as all parties had the opportunity to learn from one another. Without trust and mutual respect, the challenges may have been insurmountable. The primary challenge was time. Development and refinement of the B-SWELL materials spanned one year. It was an iterative process that required patience as the PI and C-RAB members each reviewed the materials, suggested edits, and approved changes. Another challenge came with marrying the idealistic suggestions of a community research board with the strict requirements expected from an institutional review board. Transparency was necessary so that C-RAB members understood the decisions related to everything from participant incentives to recruitment. The processes were more challenging due to COVID-19 restrictions and social distancing. Whereas meetings could be held in person pre-COVID-19, activities and interactions were required to be remote (via phone or virtually) and drafts of the B-SWELL materials were mailed or arranged for pick up. Virtual meetings could also be challenging depending on the technical abilities and network capabilities of the PI and C-RAB members.

## 4. Discussion

Using community participatory research methods and a strong working relationship between the PI and the C-RAB, content for the B-SWELL program materials was successfully developed consisting of culturally tailored, original materials and adaptations of existing AHA LS7 materials. The PI of this study approached the C-RAB with a program concept based on her prior research. However, rather than develop the program and its ideas on her own, she chose to engage with community researchers to develop a program that could be sustainable. Intertwined in this process was the desire to culturally adapt the LS7 materials to address the existing health disparities affecting midlife Black women. Community engagement and participation were essential to achieve these aims. The C-RAB consisted of community researchers that were invested in their community and representative of the targeted population, midlife Black women. Although time intensive, the cyclic process used to develop the B-SWELL materials resulted in an intervention that can be embraced by midlife Black women. The B-SWELL has unique attributes that will increase the likelihood of sustainability and transferability.

A true collaboration was formed that placed community researchers on equal footing with academic researchers. The C-RAB members provided honest and direct feedback that ranged from critique of concepts to the images of Black women and colors used in the modular design. The ideas and recommendations from the C-RAB provided rich, salient characteristics to the B-SWELL materials that embodied situations and experiences of midlife Black women. Community participatory research methods are inclusive, allowing research to be conducted with its participants rather than on its participants [15]. Ideally, community-focused research projects should arise from problems relevant to the population studied thus promoting engagement and participation [24]. When ideas for research projects arise outside of the community, such as what occurred with the B-SWELL program, the community should be engaged at the earliest point of development and refinement to improve the chance of success. The methods and approaches in community engaged research promote reflection, participation, and collaboration and are conducive to a cyclical pattern of discovery [23,24]. Community engaged research is grounded in social justice and, thus, is ideal for investigating issues associated with health disparities and high-risk or marginalized populations [24,25]. Community programs are proposed to be more sustainable and successful when developed in collaboration with representative members of the targeted community or population thus, a community-focused approach was ideal for the development and refinement of the B-SWELL program.

## 5. Conclusions

The final B-SWELL intervention modules and content were developed using community engaged and participatory research methods. The benefits of collaborative research have been recognized, yet the numbers of community-based collaborations in research projects remain small compared to the large number of researcher-led interventions and projects. Acknowledgement of the expertise derived from lived experiences and recognizing the value in the community perspective are key if researchers hope to effectively address existent health disparities and social inequities.

## Figures and Tables

**Table 1:** C-RAB Partnership Content.

Reach/Scope of the Research Statement of purpose Anticipated outcomes.
Responsibilities of the Community Partner and the Academic Partner Commitment details (roles, meetings, feedback, recruitment) Timeline
Community Relevance Products written at a 6th grade reading level or less for inclusivity Tailored to midlife Black women
Communication Expectations for informed engagement Modes of communication
Funding Source Duration C-RAB member compensation
Partnership Sustainability Intentions to maintain partnership beyond completion of project
Dissemination of Results Community spaces Academic spaces
Challenges Responsibilities should challenges arise
Statement of Acknowledgement of Partnership Agreement
Signatures of Designated C-RAB Partner and Designated Academic Partner

**Table 2:** Modular content and rationale.

Content Description	Rationale/Cultural Relevance
Objectives	Guide use of materials and provide details of what will be learned using a culturally appropriate lens.
Glossary of terms	Improve access through clarity.
Heart disease information	Increase understanding and awareness of heart disease and heart disease risk. Provide facts and statistics about Black women and heart disease.
Stress and LS7 behavior	Describe the effect of stress on heart disease risk, highlighting unique stressors experienced by Black women.
Stress reduction strategies	Provide culturally appropriate strategies for stress.
Strategies to adopt LS7	Provide culturally appropriate strategies for LS7 and information about the effect of stress on each LS7 behavior.
Case study	Illustrate challenges associated with the adoption of LS7 health behaviors through culturally appropriate storytelling.
Goal setting forms	Guide to setting realistic goals for healthy behaviors.
Images of midlife Black women	Improve appeal through culturally appropriate images.

**Table 3:** C-RAB facilitator training and corresponding duties.

Training Area	C-RAB Duties
B-SWELL modular content	Guide participants to resources and information available in the program materials.
Data collection forms	Accurate documentation of participant attendance and engagement.
Facilitation	Co-facilitation of group sessions with PI. Independent facilitation of break out rooms in Zoom.
Data Management	Confidential storage of paper documents and notes. Safe and confidential transfer of paper documents to PI. Confidential management of Zoom meeting room.
Technology	Use of tablet devices. Accessing Zoom meeting room. Management of Zoom breakout room and group participants. Management of Zoom audio recordings. Trouble shooting of common Zoom issues.

**Table 4:** Facilitator questions for reflection.

Questions for Post-Session Self-Evaluation
What do you think went well with this group session? Describe any problems you had (i.e. participant agitated, no engagement, technical difficulties, etc.) What do you think you could have done to make this session better? Describe areas where you might need more training. Describe areas for improvement.

## Data Availability

Data supporting the reported results can be found on ClinicalTrials.gov (NCT04404478).

## Appendix A

**Black Women’s Stress-Reduction Wellness Program (B-SWELL)**

**A Community Engaged Project with the West End Community Research Advisory Board (WE C-RAB)**

**Team Member Roles and Responsibilities**

**Community Partner Organization:** West End Community Research Advisory Board (WE C-RAB)

**Academic Partner Organization:** Holly Jones, PhD, RN CFNP. Assistant Professor, College of Nursing, University of Cincinnati

**Responsibilities of the Community Partner to the Academic Partner:**

1. **Purpose:** We understand that the purpose of our partnership is to collaborate to help develop the 8 week B-SWELL program prototype, as well as provide input on recruitment, site locations and marketing for the program. Additionally, at the completion of the program, we will provide feedback and recommendations about the relevance and use of the outcomes from the program and identify the best ways for findings of the program to be shared with the West End community and similar communities in the future.
2. **Reach:** We will work with our Academic Partner in the development and implementation of the B-SWELL program which will enroll 50 midlife Black women in the community. We will advocate for the program and ensure effective dissemination of the findings to the larger West End community.
3. **Commitment:** We will…
  (a) Designate one WE C-RAB member to attend all quarterly Scientific Advisory Board meetings, with one designated alternate, to problem solve and inform the project. We will encourage our representative to offer his/her opinion respectfully but confidentially and to reach out for outside support when doing so feels challenging.
  (b) Have 4–8 WE C-RAB members participate in smaller focus groups to assist in the creation of the B-SWELL program prototype.
  (c) Provide feedback about the program as well as recruitment, marketing and location at a WE C-RAB meeting in early 2020.
  (d) Advise the Academic Partner on the relevance and use of study outcome measures from the program results (satisfaction, measures of stress and depression, a person’s belief in capacity to adopt healthy behaviors and lifestyle adoption).
  (e) Identify best ways for findings of the program to be shared with the West End community and similar communities in the future.
4. **Community Relevance:** We will consider the feasibility, as well as the relevance, of this program for the West End community. We will help ensure that the program is not too complicated and is written in a manner understandable to our community, including that it is written at a 6th grade reading level or less.
5. **Timeline:** We will attempt to keep to the project timeline and share relevant information with our partners in a timely manner if commitments are unable to be kept. Likewise, we will ask them for support as we complete our roles and responsibilities on the project to stay on time.
6. **Communication:** We will…
  (a) Stay informed with the planning and follow-up to the best of our ability.
  (b) Ask questions and request information as we need it, including any concerns.
  (c) Participate in and take responsibility for the decisions/input we have agreed to provide.
  (d) Communicate in-person or by phone as appropriate and understand communication responses should be within 2 days.
7. **Funding:** We understand that awarded funds, held by the academic partner, will include compensation for Scientific Advisory Board, focus groups and WE C-RAB meetings at $20/hr in the form of a Clincard or similar payment method.
8. **Partnership Sustainability:** We understand that our partners hope to continue to work with us even after the funding period ends, and we are open to that possibility.
9. **Dissemination of Findings:** We understand that as this is a community-academic partnership, that we will need to help our academic partner report and tailor messages and materials to disseminate the project results and future action steps when the project ends (within 3 months).
10. **Challenges:** If we are not able to fulfill these commitments, we will expect that our partners will contact us about our responsibilities.

**Responsibilities of the Academic Partner to the Community Partner:**

1. **Purpose:** I understand that the purpose of our partnership is to collaborate to develop and successfully implement the 8 week B-SWELL program prototype that is relevant and feasibility to the West End community. I plan to work with community residents collaboratively, not on them.
2. **Reach:** I will work with our Community Partner in the development and implementation of the B-SWELL program which will enroll 50 midlife Black women in the community. I will provide our partners access to deidentified data and results to best communicate these results to the greater community.
3. **Commitment:** Dr. Holly Jones will be the contact researcher and her research team will attend all project meetings to co-create and implement the projects. We will encourage our entire research team to offer their opinions respectfully but confidentially and to reach out for outside support when doing so feels challenging.
4. **Community Relevance:** I will consider the feasibility and quality of the program for the West End community. I understand that the tools should not be too burdensome for residents to complete and will be written in a manner that is understandable to our target population, including that it is written at a 5th grade reading level or less.
5. **Timeline:** I will attempt to keep to the project timeline and share relevant information regarding timeline or commitment changes with all our partners in a timely manner. Likewise, I will ask my partners for support as I complete my roles and responsibilities on the project to stay on time.
6. **Communication:** I will…
  (a) Stay informed with the planning and follow-up to the best of our ability.
  (b) Ask questions and request information as we need it, including in cases of any concerns.
  (c) Participate in and take responsibility for the decisions/input we have agreed to provide.
  (d) Communicate in-person or by phone as appropriate and understand communication responses should be within 2 days.
7. **Funding:** I understand that awarded funds, held by the academic partner, will include compensation for Scientific Advisory Board, focus groups and WE C-RAB meetings at $20/hr in the form of a Clincard or similar payment method.
8. **Partnership Sustainability**: I hope to continue to work with my Community Partners even after the funding period ends, and I will work to encourage new ideas and action steps as a result of this project.
9. **Dissemination of Findings:** I understand that I will need to tailor my messages and materials to disseminate the project’s results and future action steps when the project ends (within 3 months).
10. **Challenges:** If I am not able to fulfill these commitments, I will expect that my partners will contact me about my responsibilities. Depending on the situation, we may be asked to remedy unmet commitments.

**Acknowledgement:** We hereby sign acknowledging the roles and responsibilities as outlined above. We understand that roles, responsibilities and details of expectations may be added as the project evolves.

**Representative Signatures**

______________ ________

**Name of Designated Community Partner(s) Signature Date**

**__________________ ____________________  ________________ ______**

**Name of Designated Community Partner(s)  Signature Date**

**__________________ ____________________  ________________ ______**
